# Nine Cluster E mycobacteriophages isolated from soil

**DOI:** 10.1128/mra.00463-24

**Published:** 2024-08-30

**Authors:** Joseph M. Gaballa, Amanda Freise, Krisanavane Reddi, Jordan Moberg Parker

**Affiliations:** 1 Department of Medicine, University of Colorado Anschutz Medical Campus, Aurora, Colorado, USA; 2 Department of Microbiology, Immunology, and Molecular Genetics, University of California, Los Angeles, Los Angeles, California, USA; 3 Department of Biomedical Science, Kaiser Permanente Bernard J. Tyson School of Medicine, Pasadena, California, USA; Loyola University Chicago, Chicago, Illinois, USA

**Keywords:** mycobacteriophage, bacteriophages

## Abstract

Mycobacteriophages FireRed, MISSy, MPhalcon, Murica, Sassay, Terminus, Willez, YassJohnny, and Youngblood were isolated from soil using *Mycobacterium smegmatis* as a host. Genome sequencing and annotation revealed that they belong to Actinobacteriophage Cluster E. Here, we describe the features of their genomes and discuss similarities within these Cluster E phages.

## ANNOUNCEMENT

The discovery of mycobacteriophages is typically conducted using *Mycobacterium smegmatis*, a non-pathogenic strain that serves as a model host for pathogenic mycobacteria. Over 150,000 genes have been identified, a majority of which have unknown function, in the collection of over 2,000 mycobacteriophages, which have been sequenced (1, 2). This incredible degree of genetic diversity warrants mycobacteriophages to be organized into clusters and subclusters based on genomic similarity (3). In this study, we introduce the genomes of nine Cluster E mycobacteriophages isolated from soil by undergraduates in the Science Education Alliance-Phage Hunters Advancing Genomics and Evolutionary Science (SEA-PHAGES) program (4).

Soil samples were collected from sites around the greater Los Angeles, CA, area, and phages were isolated using direct or enrichment isolation (Table 1) on *M. smegmatis* MC^2^155 at 35°C as described by the SEA-PHAGES Discovery Guide (5). DNA was isolated using the Wizard Promega DNA Clean-Up Kit (#A7280). Pooled libraries were prepared with a NEB Ultra II DNA Library Prep kit (NEB #E7103) for Illumina sequencing or a Roche GS FLX Titanium emPCR Lib-A Kit for 454 GS FLX sequencing (Table 1). Sequence reads were assembled into single-phage contigs using Newbler v2.9 (454 Life Sciences) with default settings and assessed for completeness and genomic termini using Consed v29 as previously described (6, 7). Location and coding potential of putative genes were predicted using DNA Master [J. G. Lawrence lab (http://cobamide2.bio.pitt.edu)], which integrates both Glimmer and GeneMark to detect potential open-reading frames (8, 9). Location calls were curated using Phamerator and Starterator (10). ARAGORN and tRNAscan-SE were used to detect the presence of tRNA genes (11, 12). Functional calls were predicted using the PhagesDB and NCBI databases, including the Conserved Domain Database, HHPred, and TMHMM (13–17). Gene Content Similarity (GCS) was calculated using PhagesDB (https://phagesdb.org/genecontent/) (18). Unless otherwise stated, no modifications were made to kit instructions, and the current version of each software package at the time of isolation was used with default parameters.

**TABLE 1:** Isolation, sequencing, and genomic features of the Cluster E phages

Mycobacteriophage	Isolation method	Collection year	Sample location (Lat, Lon)	Plaque morphology and diameter	Genome length (bp)	No. of genes	GC content (%)	3′ Overhang Sequence	Sequencing method	No. ofreads	Avg. spot length(bp) (SD)	Sequencing coverage	Sequence read archive accession no.	GenBank accession no.
FireRed	Enriched	2013	34.08 N 118.40 W	Turbid (4 mm)	76,217	150	63.0	CGCTTGTCA	Roche 454 GS FLX	8,421	512 (51.2)	48 x	SRX23607957	MF919506
MISSy	Enriched	2014	34.056 N 118.442 W	Bullseye (3.5–6.0 mm)	75,808	147	63.1	CGCTTGTCA	Roche 454 GS FLX	11,565	514 (53.5)	63 x	SRX23607958	MF919524
MPhalcon	Direct	2017	33.9922 N 118.4705 W	Clear/halo (2–4 mm)	75,605	148	63.1	CGCTTGTCA	Illumina MiSeq 150-base single-end reads	1,090,304	146 (15.0)	2,029 x	SRX23702566	MH020247
Murica	Enriched	2013	34.07 N 118.451 W	Clear/bullseye(3–5 mm)	77,053	149	63.0	CGCTTGTCA	Roche 454 GS FLX	14,503	506 (61.1)	81 x	SRX23607959	MF919525
Sassay	Enriched	2014	34.0703 N 118.453 W	Turbid with halos (4 mm)	73,495	141	63.0	CGCTTGTCA	Roche 454 GS FLX	22,872	506 (58.8)	126 x	SRX23607960	MF919529
Terminus	Enriched	2014	34.0675 N 118.45444 W	Clear (2–4 mm)	76,169	149	63.1	CGCTTGTCA	Illumina HiSeq 150-base paired reads	17,911,466	98 (9.2), 98 (9.3)	10,582 x	SRX23607963	MF919535
Willez	Enriched	2011	34.021 N 118.395 W	Turbid (5 mm)	74,576	144	62.9	CGCTTGTCA	Roche 454 GS FLX	24,148	512 (50.1)	157 x	SRX23607961	MF919540
YassJohnny	Enriched	2015	34.0656 N 118.4540 W	Turbid/Bullseye(2 mm)	73,697	141	62.9	CGCTTGTCA	Illumina MiSeq 150-base single-end reads	516,974	127 (22.0)	913 x	SRX23702568	MF919541
Youngblood	Enriched	2014	34.075 N 118.451 W	Turbid/bullseye(2–5 mm)	75,896	150	62.9	CGCTTGTCA	Roche 454 GS FLX	6,987	511 (55.5)	38 x	SRX23607962	MG099953

**Fig 1:**
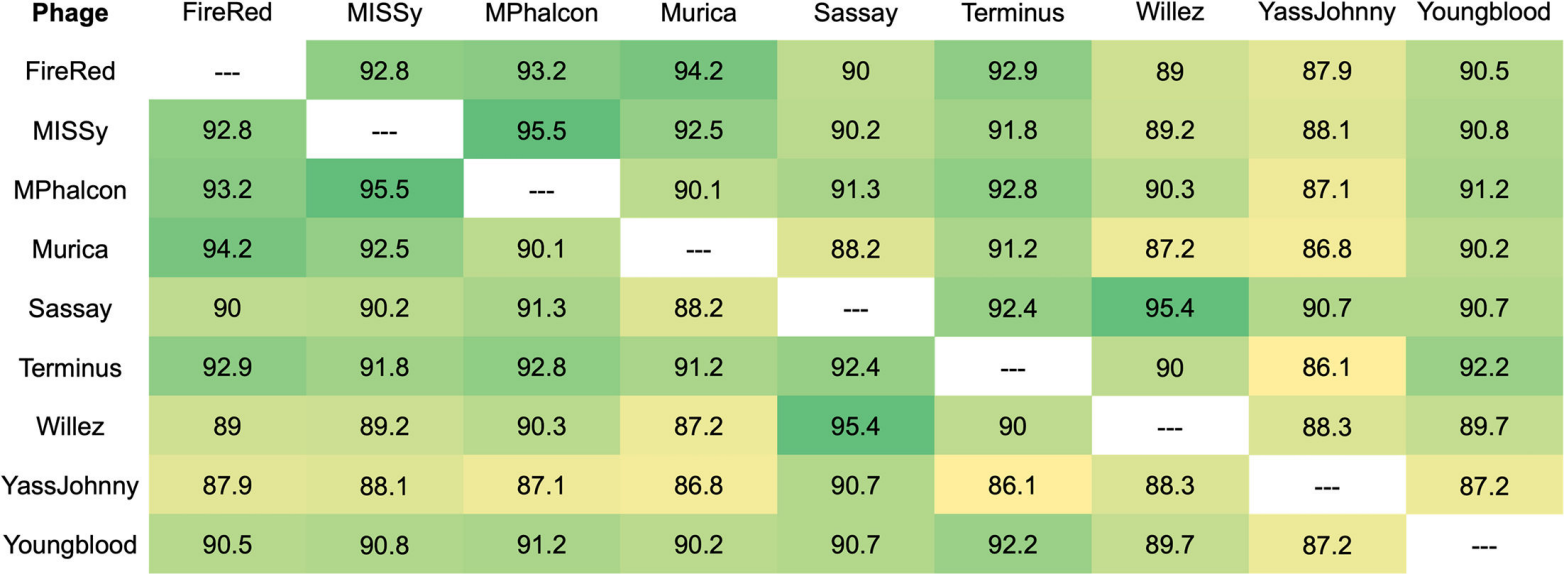
Gene Content Similarity (GCS) of the Cluster E phages. GCS, which is a calculation of the average number of shared genes between two phages, ranged from 86.1% to 95.5% (18). The GCS calculated for each phage pair is presented as a heat map with the highest GCS scores represented in dark green and the lowest GCS scores represented in yellow.

Local whole-genome BLASTn against the Phagesdb.org database (https://phagesdb.org/blast/) indicated 98%–99% identity of all nine phages with previously characterized Cluster E phages. Complete genome lengths ranged between 73,495 and 77,053 base pairs with each genome containing a 3′ sticky overhang (CGCTTGTCA). GC content ranged from 62.9% to 63.1%, and the average of 63.0% was consistent with the average of published Cluster E phages (63.0%). Total predicted gene counts ranged between 141 and 150 (Table 1), with pairwise GCS ranging between 86.1% and 95.5% (Fig. 1). Most of the genes with no annotated functions are located on the right side of the genome, while the left side of the genome is highly conserved and contains most of the structural proteins. Each phage genome contains two tRNAs and a lysis cassette comprising lysin A, lysin B, and holin genes. Integrase and an immunity repressor are found downstream of the lysis cassettes in all phages. The presence of a lysis cassette, integrase, and an immunity repressor suggests that these phages can undergo lytic or lysogenic life cycles, and this was supported by the presence of both clear and turbid or bullseye plaque morphologies.

## Data Availability

The Whole Genome Sequencing reads and Complete Genome sequences have been deposited in the NCBI Sequence Read Archive (SRA) and GenBank, respectively (Table 1). The versions described in this paper are the first versions.
